# Collembase: a repository for springtail genomics and soil quality assessment

**DOI:** 10.1186/1471-2164-8-341

**Published:** 2007-09-27

**Authors:** Martijn JTN Timmermans, Muriel E de Boer, Benjamin Nota, Tjalf E de Boer, Janine Mariën, Rene M Klein-Lankhorst, Nico M van Straalen, Dick Roelofs

**Affiliations:** 1 Vrije Universiteit, Institute of Ecological Science, Department of Animal Ecology, De Boelelaan 1085, 1081 HV, Amsterdam, The Netherlands; 2 PRI Greenomics, Droevendaalse steeg 1, 6708 PB Wageningen, The Netherlands

## Abstract

**Background:**

Environmental quality assessment is traditionally based on responses of reproduction and survival of indicator organisms. For soil assessment the springtail *Folsomia candida *(Collembola) is an accepted standard test organism. We argue that environmental quality assessment using gene expression profiles of indicator organisms exposed to test substrates is more sensitive, more toxicant specific and significantly faster than current risk assessment methods. To apply this species as a genomic model for soil quality testing we conducted an EST sequencing project and developed an online database.

**Description:**

Collembase is a web-accessible database comprising springtail (*F. candida*) genomic data. Presently, the database contains information on 8686 ESTs that are assembled into 5952 unique gene objects. Of those gene objects ~40% showed homology to other protein sequences available in GenBank (blastx analysis; non-redundant (nr) database; expect-value < 10^-5^). Software was applied to infer protein sequences. The putative peptides, which had an average length of 115 amino-acids (ranging between 23 and 440) were annotated with Gene Ontology (GO) terms. In total 1025 peptides (~17% of the gene objects) were assigned at least one GO term (expect-value < 10^-25^). Within Collembase searches can be conducted based on BLAST and GO annotation, cluster name or using a BLAST server. The system furthermore enables easy sequence retrieval for functional genomic and Quantitative-PCR experiments. Sequences are submitted to GenBank (Accession numbers: EV473060 – EV481745).

**Conclusion:**

Collembase  is a resource of sequence data on the springtail *F. candida*. The information within the database will be linked to a custom made microarray, based on the Agilent platform, which can be applied for soil quality testing. In addition, Collembase supplies information that is valuable for related scientific disciplines such as molecular ecology, ecogenomics, molecular evolution and phylogenetics.

## Background

Organisms are able to maintain homeostasis in changing environments by regulating their metabolic machinery. To accomplish this, organisms continuously have to adjust the expression of their genes. This is particularly evident when environmental challenges drive organisms to the boundaries of their ecological niche and induce stress responses (e.g. [1]). In recent years, significant understanding has been obtained on the signal transduction pathways by which stress affects gene transcription [2]. The question arises whether it is possible to sense aspects of the environment by investigating transcriptional profiles of exposed organisms.

Recent advances in the field of toxicogenomics suggest that environmental quality can indeed be diagnosed by transcriptional profiling [3] and it is generally acknowledged that genomic techniques, and more specifically transcriptomics, have the potential to revolutionize environmental risk assessment [4-9]. The prospects are that gene expression studies will enable a fast and sensitive detection and evaluation of environmental stressors and toxicants. This is strengthened by the fact that several recent studies have shown that transcription profiling can be applied as an early indicator of toxicity [10,11] in a dose-dependent manner [12].

We started a project that aims to develop a microarray-based methodology for soil quality assessment using the parthenogenetic springtail *Folsomia candida *(Collembola). This species, which is easy to culture and has a short generation time, was chosen because it is already a standard test organism in ecotoxicology [13]. It lives in direct contact with the soil and toxicological data are already widely available (e.g. ECOTOX database from U.S. EPA [14]). Furthermore, a standard test looking at survival and reproduction after 28 day exposure is in place that follows OECD (Organisation for Economic Co-operation and Development) and ISO (International Standard Organization) guidelines. Although the latter test is conducted in a standardized laboratory setting, it has been shown that the outcomes are predictive of natural situations [15]. However, there are several shortcomings to the current test. First, it does not provide information about the nature of the stressor. Second, the mode of action of toxicants cannot be verified. Third, the test is time-consuming as it lasts for at least 28 days. Finally, the test is rather labor intensive.

By extending the ISO standard test with genomic technologies, these shortcomings may be circumvented. However, genomic information on *F. candida *is very poor: a search for sequences yields only 52 hits in the National Center of Biotechnology (NCBI;[16]) nucleotide database (July 5^th ^2007), mainly consisting of 18S rRNA, 28S rRNA and cytochrome c oxidase sequences used as phylogenetic markers.

A time- and cost effective way to retrieve sequence information on the functional part of the genome is to set up an Expressed Sequence Tag (EST) project, which was conducted for the *F. candida *transcriptome. Here we report on the sequencing and annotation of ~9000 ESTs, which form the starting point for the construction of an oligo array that can be applied in soil quality testing. The sequences were processed, assembled, BLAST-based annotated and stored in a web-accessible database [17]. The database can be searched for BLAST-based annotations and Gene Ontology terms [18] and by using a stand alone BLAST server. Collembase furthermore enables retrieval of sequence information on (differentially) expressed genes, which can then be applied in functional genomic and Quantitative-Polymerase Chain Reaction (Q-PCR) validation experiments.

Although Collembase was primarily created for the development of a microarray, we expect that it is of interest for researchers outside the field of ecotoxicology as well. Due to its short generation time, *F. candida *is often used in ecological studies [13]. In addition, Collembola have a crucial position in the phylogeny of the arthropods and, thus, also have the attention from evolutionary biologists (e.g. [19]). The retrieved genome data will significantly enhance molecular ecological and evolutionary studies on *F. candida*.

## Construction and content

### Construction of cDNA libraries

To restrict redundant sequencing we chose to start our EST project with a normalized cDNA pool. RNA extraction from the parthenogenetic, clonally reproducing collembolan *Folsomia candida *(laboratory strain 'Berlin'; Vrije Universiteit Amsterdam) was carried out using the Spin Vacuum (SV) Total RNA isolation system (Promega). Animals (eggs, juveniles and adult females) were taken from a culture of mixed age with a more or less even age distribution. All animals (~100 mg) were pooled before RNA extraction. Concentration and purity of the total RNA pool was checked by UV absorption (260 and 280 nm). Quality of total RNA was evaluated on a 1% agarose gel (stained with SYBR Gold stain; Invitrogen) and on an Agilent BioAnalyzer (Agilent Technologies). Afterwards 0.1 volumes of 3 M sodium acetate and 3 volumes of 96% ethanol were added and total RNA was shipped at room temperature to Evrogen (Moscow, Russia).

Double-stranded cDNA synthesis (SMART technology [20]), normalization and library construction were performed by Evrogen. The reaction was started with 0.3 μg total RNA and cDNA was SMART amplified (18 PCR cycles) and normalized by the procedure described by [21], which consists of cDNA denaturation/reassociation, a duplex-specific nuclease (DNS) treatment [22] and PCR amplification. The cDNA thus obtained was used for library construction as follows. The cDNA was incubated with restriction enzymes Sbf1 and Not1, and ligated into Sbf1 and Not1 digested pAL17.2 vector (Evrogen). The resulting plasmids were subsequently transformed into *E. coli *(Evrogen). Finally, glycerol stocks were made (17% glycerol), which were transferred to the Vrije Universiteit (Amsterdam) on dry-ice and stored at -80°C until further use.

Efficiency of the procedure was examined by determining the abundance of several transcripts before and after normalization using Q-PCR. Primers were developed based on five available GenBank accessions and β-actin. Genes amplified were β-actin (GenBank:EU037094), USP-RXR (GenBank:AY157930), Ultrabitorax (GenBank:AF435789), Kruppel (GenBank:AF395109), RNA helicase Dead1 (GenBank:AY043229) and 28S rDNA (GenBank:AF483424). Primer sequences are given on [17] (see Additional file 1). Primers were developed using Primer Express version 1.5 (Applied Biosystems Inc., Foster City, USA), using the following parameters: Minimum Tm: 59–60°C, Maximum Tm difference between primers: 1°, Oligo length: 20–25 bp, Amplicon length: 90–120 bp.

**Figure 1:**
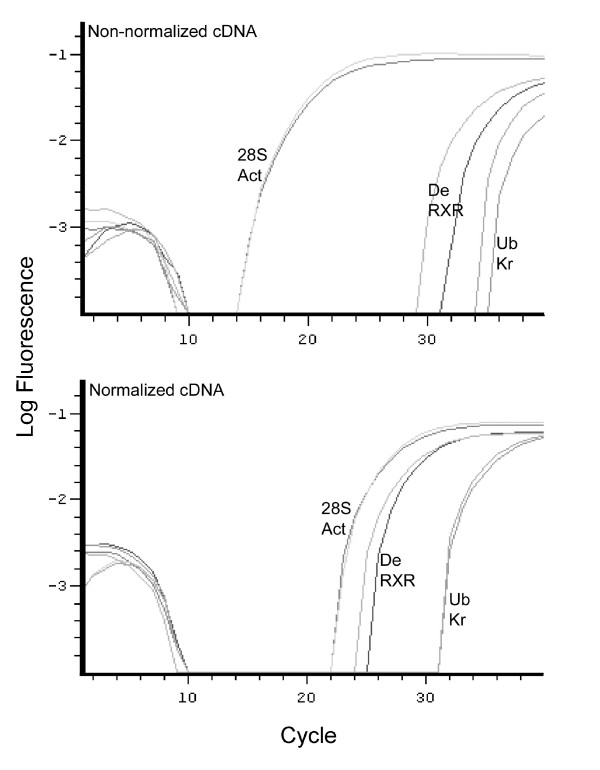
Relative abundance of six cDNAs before (upper) and after (lower) normalization as measured using quantitative PCR. Act: β-actin; 28S: 28S rDNA; De: RNA helicase Dead1; RXR: RXR-USP; Ub: Ultrabithorax; Kr: Kruppel.

Real-time PCR was performed on an Opticon 1 real-time PCR machine (MJ Research) using SYBR green 2X Mastermix (Finnzymes), according to [23]. Real time PCR reactions used 3 μl normalized and non-normalized non-ligated cDNA template (0.2 μg/100μl). The program used for amplification was: denaturation (95°C for 15 min.), 2-step amplification and quantification (92°C for 15s, 60°C for 1 min. and one fluorescence measurement), melting curve program (60–90°C with a heating rate of 0.1°C per second and one fluorescence measurement per second). As can be seen in Figure 1 the normalization procedure was effective: transcripts that were highly abundant in the original pool (Figure 1A) occurred considerably diminished after normalization (Figure 1B) as compared to lower abundant transcripts. Differences in Ct-values between the high abundant 28S rRNA and β-actin transcripts and the less abundant USP-RXR and RNA helicase Dead1 transcripts was reduced from about 14 cycles to less than three cycles. However, the least abundant transcripts (Ultrabithorax and Kruppel) were not very well enriched: they maintained high Ct-values.

De Boer et al. (unpublished data) constructed cDNA libraries enriched for stress responsive genes as described by [24]. In short, 960 clones were isolated from each of two subtracted cDNA libraries enriched for 1) cadmium- and 2) phenanthrene responsive genes. Both libraries were built using the suppression subtractive hybridization procedure (SSH) [25] making use of poly (A)+ RNA isolated from ~150 exposed unsynchronized adult individuals (whole body; laboratory strain 'Berlin'; Vrije Universiteit Amsterdam). Exposure to cadmium was performed by placing animals on cellulose filters wetted to approximately 50% water-holding capacity with a 267 μmole/l CdCl_2 _solution for 48 h. Animals were exposed to phenanthrene by placing them on a compressed layer of LUFA 2.2 soil spiked with 840 μm/kg phenanthrene according to the standard ISO11267 [26] protocol for 6 days.

### EST sequencing, bioinformatics and construction of the database

In total, 9984 cDNA clones were picked and sequenced (Greenomics; Wageningen University and Research Center) using the M13 forward primer. Clones originating from the normalized library were sequenced from the 5' end of the gene (8064 total). The cDNA fragments from the SSH procedure were not ligated directionally, and therefore not sequenced from a predefined orientation (960 clones from each of the two libraries).

Raw trace files were processed using Trace2dbest [27], employing a Phred [28,29] quality threshold of 20 and a minimal high quality sequence length of 150 base pairs (bp). Of the 9984 sequences 1142 sequences did not pass the quality control, and were excluded from further analysis. A summary of the number of sequences that remained from each of the three libraries after processing of the raw data is given in Table 1.

**Table 1:** Remaining sequences after the Trace2dbest process

Library	# Clones sequenced	# Passed (%)
Normalized	8064	7329 (91)
Cadmium enriched	960	705 (73)
Phenanthrene enriched	960	808 (84)

Total	9984	8842 (89)

CLOBB [30] and Phrap (P. Green, personal communication [31]) were applied, as part of the Partigene script [27], to cluster and assemble the ESTs into unique gene objects. This procedure resulted in 6092 unique sequences. There were 4686 singletons and 1406 clusters with more than one sequence. Of those 1406 clusters 920 consisted of two sequences only. The redundancy (defined as total number of sequences/clusters) was 1.45, 1.32 and 1.62 for the total dataset, the normalized library and the cadmium library respectively, but appeared considerably higher in the phenanthrene enriched library (3.18). The highest sequence depth also occurred the phenanthrene enriched library with 98 ESTs in one cluster, compared to a maximum of 31 and 16 ESTs per cluster for the normalized and cadmium library respectively.

Sequences that were assigned to one cluster were not always assembled into one single contiguous consensus sequence (contig) by Phrap, due to high quality base pair differences between sequences. The Phrap assembly (Partigene default criteria) resulted in a total number of 6212 contigs instead of the 6092 given above (Table 2). The length of those 6212 contigs ranged between 153 bp and 1636 bp and was on average 520 bp (see Additional file 2). The sequence variation that was observed within those clusters might constitute natural occurring (allelic) variation (e.g. Single Nucleotide Polymorphisms), Taq polymerase errors and/or gene duplications, and will have to be confirmed by re-sequencing efforts.

**Table 2:** Contigs per cluster, as generated by CLOBB and Phrap

	# Clusters	# Contigs/cluster	Total number of contigs
Clusters	1	11	11
	1	10	10
	1	5	5
	7	3	21
	83	2	166
	1313	1	1313*
Singletons	4686	1	4686

Total	6092		6212

* In 75 instances Phrap did not assemble the contigs, in those cases the pseudo-contig files generated by PartiGene were used.

**Figure 2:**
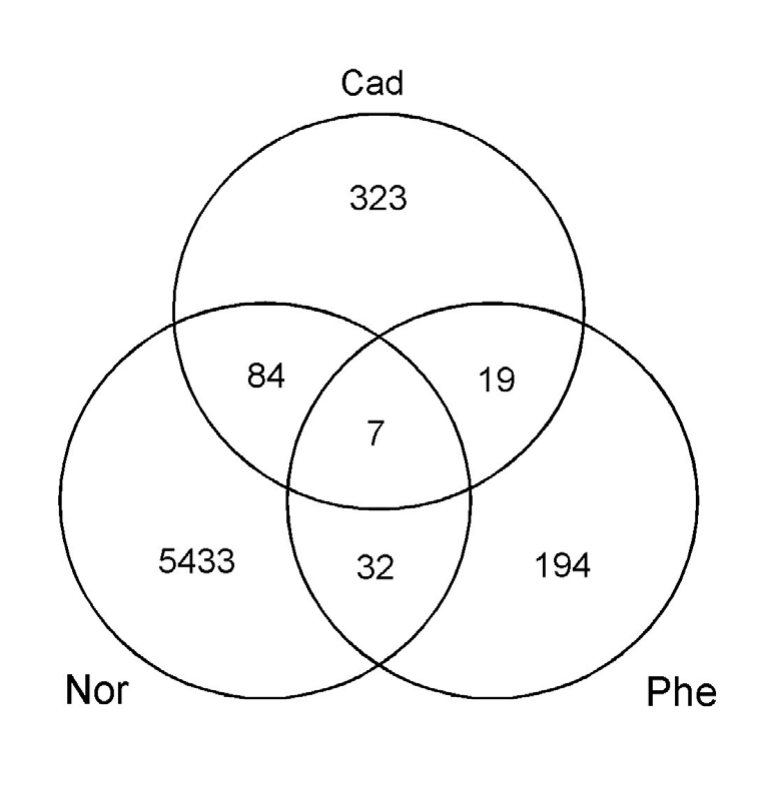
Venn-diagram showing the cluster overlap between the three libraries for the total dataset: Cad: cadmium enriched library; Phe: phenanthrene enriched library; Nor: normalized library.

Furthermore, a PERL script, which is made available on [17], was used to determine the sequence overlap between the three libraries. This script determined for each cluster which library contributed ESTs to that cluster. The overlap appeared rather low (Figure 2). Only seven clusters contained sequences from each of the three libraries (Table 3). At least three of those clusters remained un-annotated. However, it has to be mentioned that the sequence overlap that was observed might be an underestimation of the actual overlap in the database, as 5' sequencing (Normalized library) generally results in an overestimation of the number of unique sequences [32].

**Table 3:** A) Clusters that contain sequences from all three libraries and B) the most abundantly sequenced transcripts for each of the three *F. candida *cDNA libraries. n = the number of sequences that are found in a cluster and that originate from the library specified. e-values for blast analyses against 'nr'-databases

Library	Cluster (n)	Overview of related sequences (blastx)	Species	GenBank Accession	blastx e-value
A.					
All three	Fcc00101 (5)	Hypothetical protein	*Caenorhabditis elegans*	CAA90252	1e-32
		BCS1-like	*Mus musculus*	AAH19781	3e-29
	Fcc02080 (3)	No Significant Hit	-		-
	Fcc00256 (6)	No Significant Hit	-		-
	Fcc00343 (22)	Hypothetical protein	*Aspergillus nidulans*	XP_001397474	1e-19
		Haloacid dehalogenase-like hydrolase	*Neosartorya fischeri*	XP_001260321	2e-19
		Hypothetical protein	*Danio rerio*	NP_001017717	8e-06
	Fcc01457 (8)	Cytochrome c oxidase s.u.II	*Folsomia candida*	AAS66294	7e-93
	Fcc03109 (3)	No Significant Hit	-		-
	Fcc00170 (27)	Alpha-aminoadipyl-cysteinyl -valine synthetase	*Lysobacter lactamgenus*	BAA08846	4e-58

B.					
Normalized	Fcc00179 (31)	No Significant Hit	-		-
	Fcc00087 (25)	No Significant Hit	-		-
	Fcc00164 (16)	No Significant Hit	-		-
	Fcc00632 (14)	No Significant Hit	-		-
	Fcc00225 (12)	GA19585-PA	*Drosophila pseudoobscura*	EAL32218	2e-06
Phenanthrene	Fcc00058 (98)	Dipeptidyl peptidase	*Nasonia vitripennis*	XP_001607433	9e-35
		Cytochrome P450	*Aedes albopictus*	AAF97937	1e-12
	Fcc00015 (91)	Cytochrome P450	*Anopheles minimus*	AAN05727	9e-15
	Fcc00021 (35)	Monooxygenase, DBH-like 1	*Rattus norvegicus*	AAH91331	1e-21
	Fcc00217 (25)	Monooxygenase, DBH-like 1	*Gallus gallus*	NP_989955	2e-08
	Fcc04217 (23)	Cytochrome P450	*Apis melifera*	XP_392000	3e-12
Cadmium	Fcc01017 (16)	Hypothetical protein	*Ustilago maydis*	XP_757859	5e-13
		Endo-1,3 1,4-beta-D -glucanase precursor	*Oryza sativa*	XP_480878	2-07
	Fcc00170 (15)	Alpha-aminoadipyl-cysteinyl -valine synthetase	*Lysobacter lactamgenus*	BAA08846	4e-58
	Fcc01428 (16)	16S ribosomal RNA gene	*Folsomia candida*	AY555551	1e-66*
	Fcc01142(12)	No Significant Hit	-		-
	Fcc00018 (9)	No Significant Hit	-		-

*) e-value from blastn

The contigs were subjected to BLAST [33] searches of GenBank using blastx (against non-redundant database), blastn (against non-redundant database), tblastx (against dbEST) and an additional blastx (against non-redundant database restricted to Insecta). In addition, sequences were compared to all known and predicted proteins of *Caenorhabditis elegans, Drosophila melanogaster *and *Mus musculus*. Those species were chosen as they have fully sequenced genomes. In addition, *C. elegans *and *D. melanogaster *belong, like *F. candida*, to the group of molting animals (Ecdysozoa). A summary of the BLAST analyses is given in Table 4. Clusters that were perfect nucleotide matches to baker's yeast (*Saccharomyces cerevisiae*; 125 clusters) and human sequences (15 clusters) were regarded as contamination and later on removed. The relatively high number of yeast clusters observed (~2%) is explained by the fact that in our laboratory *F. candida *is fed baker's yeast. The fact that the food of *F. candida *is in itself a genomic model species was advantageous when pruning the database: these sequences are readily identified by their high bit and e-values scores in the BLAST searches.

**Table 4:** Percentages of contigs showing sequence similarity (e-value < 10^-5^) with sequences stored in GenBank (nr, est databases and nr database restricted to the Insecta) and proteins of *Caenorhabditis elegans, Drosophila melanogaster *and *Mus musculus *(April 2007)

Database	BLAST	Significant hits for the total dataset	Significant hits excl. 140 clusters*
nr	blastx	42	41
nr	blastn	9	7
est	tblastx	40	39
nr – Insecta**	blastx	36	35
*C. elegans*	blastx	25	24
*D. melanogaster*	blastx	32	30
*M. musculus*	blastx	31	29

* In total 140 clusters showed high similarity to yeast and human DNA sequences stored in GenBank and were therefore regarded as contamination.

** Blast analysis performed August 2007

*F. candida *harbors intracellular bacteria of the genus *Wolbachia *[34] and its gut contains many bacterial species as well [35]. Those might turn up as contaminating sequences in the EST dataset. To pinpoint contaminating sequences from bacterial origin the clusters were compared to all protein encoding sequences found in the genome of *Escherichia coli *(GenBank: U00096) and in the *Wolbachia *endosymbiont of *Drosophila melanogaster *(GenBank: AE017196). Sequences showing significant homology to *E. coli *or *Wolbachia *(blastx; e-value < 10^-5^), but not to *D. melanogaster, C. elegans or M. musculus*, were marked as putative contaminants. In total 70 of such clusters were retrieved, which overlapped to a great extent (56 *E. coli *and 32 *Wolbachia *clusters): In total 18 clusters appeared in both analyses (see Additional file 3). Those putative 'bacterial clusters' were not excluded from further analysis, as our procedure does not guarantee if a sequence is contamination or not.

Table 3 shows the five most abundant transcripts for each of the three libraries. The SSH procedure conducted on phenanthrene exposed animals appeared efficient. Of the top five phenanthrene clusters three show high similarities to monooxygenases of the cytochrome P450 enzyme family, which are known to be involved in phase I biotransformation of lipophilic substances such as phenanthrene [36]. The two other clusters show homology to other monooxygenases, and might be involved in phase I metabolism as well. The results for the cadmium library are less straightforward. Two of the five most abundant clusters remain un-annotated, and two clusters show resemblance to accessions that are not from animal origin. Note that one of those two latter clusters (cluster Fcc00170) occurred in all three libraries (Table 3). As with the 'bacterial clusters', those clusters are currently not discarded from the database and are submitted to GenBank. Supplementary experiments will be conducted to determine the exact origin of those clusters, and whether or not they represent contaminants.

The absence of highly expressed house-keeping genes among the five most abundant transcripts in the normalized library, suggests that the normalization procedure was successful. Without normalization more highly abundant transcripts, like tubulins, ribosomal proteins and actins, would have been sequenced (e.g. [37]). Although these sequences are present in the dataset, they do not form the list of most abundantly sequenced transcripts. For example, more than 40 ribosomal protein sequences were obtained (e.g. cluster Fcc02740), but most of these were represented by only one or two ESTs.

The prot4EST [38] script was applied to infer protein sequences (excluding the DECODER program). Putative open reading frames of the total dataset ranged between 23 and 440 amino-acids, and had an average length of 115 amino-acids. The amino acid sequences were annotated with Gene Ontology terms (GO; ) using the PartiGene [27] annot8r_blast2GO script (Schmid and Blaxter, personal comm.; [39]). An overview of the results of these analyses is given in Table 5. Of the 6212 contigs 1126 contigs (~18%) were assigned at least one GO term (expect-value < 10^-25^; 1025 contigs when excluding the 140 clusters originating from yeast and human mRNA from the analysis). The Partigene [27] PERL scripts were used to store all the information in a web-accessible relational database [17]. All processed ESTs, excluding the ones marked as human and yeast contamination, were submitted to dbEST (accession numbers:  – ).

**Table 5:** GO slim terms for *F. candida *genes based on a BLAST search (e-value < 10^-25^) against the GO annotated UniProt database as generated by Annot8r_blast2GO

**Description**	**Gene Ontology ID**	**Occurrences**
**Biological process**		

Electron transport	GO:0006118	53
Response to stimulus	GO:0050896	18
Amino acid and derivative metabolism	GO:0006519	31
Behavior	GO:0007610	1
Physiological process	GO:0007582	500
Transport	GO:0006810	140
Regulation of biological process	GO:0050789	2
Cell communication	GO:0007154	38
Nucleobase, nucleoside, nucleotide and nucleic acid metabolism	GO:0006139	158
Cell motility	GO:0006928	3
Development	GO:0007275	30
Cellular process	GO:0009987	6
Biological process unknown	GO:0000004	3

**Molecular function**		

Motor activity	GO:0003774	8
Transcription regulator activity	GO:0030528	8
Antioxidant activity	GO:0016209	2
Signal transducer activity	GO:0004871	16
Enzyme regulator activity	GO:0030234	15
Catalytic activity	GO:0003824	571
Binding	GO:0005488	543
Nucleic acid binding	GO:0003676	128
Molecular function unknown	GO:0005554	31
Structural molecule activity	GO:0005198	82
Transporter activity	GO:0005215	65

**Cellular component**		

Extracellular region	GO:0005576	16
Intracellular	GO:0005622	444
Unlocalized protein complex	GO:0005941	3
Cellular component unknown	GO:0008372	2
Cell	GO:0005623	200

## Utility and discussion

### Current contents of the database

Currently, Collembase comprises data on 8686 ESTs, which are structured in 5952 clusters. That is 6092 minus the 140 clusters from yeast and human origin. To enable easy access to the sequence dataset, the information gathered was stored in a relational database and a web-interface was created. For all clusters data is offered on (1) the ESTs within a cluster and their clone names, (2) the cDNA library from which the ESTs originated, (3) blastx and blastn hits against GenBank 'nr' databases and tblastx hits against dbEST, which all will be updated regularly, (4) the consensus sequences as generated by Phrap, and (5) the GO terms when available. Furthermore, for each cluster the BLAST results and the processed ESTs can be downloaded.

Collembase can be explored library-specific using text queries (e.g. cluster name or BLAST annotation) and by sequence similarity using a local BLAST [33] server. Furthermore, a Primer3 web-server [40] was implemented to enable PCR primer design on the assembled sequences.

### Future application and intended uses of the database

#### Soil quality and risk assessment

The dataset presented here was generated mainly to obtain the required genomic information to construct a microarray for soil quality assessment. The array, which is based on the Agilent microarray technology, is linked to Collembase: The 60-mer oligos printed on the chip follow the nomenclature of the clusters from which they were derived. This "linkage" enables straightforward sequence retrieval. Sequences of differentially expressed genes can be downloaded from Collembase and used in validation experiments (e.g. Q-PCR). Furthermore, in the near future we intend to store microarray and Q-PCR gene expression data as well. This freely accessible online repository will allow evaluation and analysis of the data by the scientific community (*sensu *[41]).

The small overlap between the toxicant enriched libraries and the normalized library (Figure 2), in combination with the higher redundancy of the toxicant enriched libraries (especially the phenanthrene library), suggests that metal and PAH exposure trigger different genes in *F. candida*. Although our expression data on *F. candida *still have to be verified by actual gene expression assays, such specificity would imply that transcription profiles contain a signature of the nature of the stress, and that different stresses can be distinguished by transcription profiling. This view is strengthened by a recent ecotoxicogenomic study by [42]. These authors showed that in the crustacean *Daphnia magna *different substances belonging to one chemical class (metals) can be discriminated on the basis of their characteristic expression profiles. Finally, we believe that transcription profiling will enable mechanistic insight in responses to mixtures of toxicants, a relatively new and unknown field in (eco)toxicology.

## Other applications

The collembolan *F. candida *is frequently used in experimental studies (for a recent review see [13]), therefore Collembase could be useful outside the field of ecotoxicology as well. We expect applicability in the following research areas:

### Ecogenomics

To fully disentangle the molecular mechanisms by which organisms deal with ecological challenges and environmental stress, additional ecologically relevant model organisms are needed [36,43]. *F. candida *is among a few others (e.g. free-living nematodes and earthworms [37,44]) one of the first soil organisms that is subject to EST sequencing. Collembase could form the basis of *F. candida *becoming a model organism in the research field of ecogenomics.* F. candida *has this potential as the species is easy to rear in the laboratory, reproduces parthenogenetically, has a short generation time, has a well-defined ecology and is traceable in (mesocosm) field experiments. It seems obvious that the sequence information stored in Collembase can be exploited to answer ecological questions, e.g. related to drought-tolerance, starvation and microbial resistance in soil ecosystems.

### Molecular ecology and population genetics

The EST dataset presented holds information applicable in molecular ecological- and population genetic studies. For example, within the dataset 184 contigs showing one or more tandem-repeats (microsatellites) with a minimum of five repeats were discovered using the MISA PERL script [45] (Additional file 4). Within some of the clusters up to three different alleles were observed. However, due to the limited redundancy in our dataset and the fact that the libraries were constructed from animals from one parthenogenetic strain it is impossible to determine their degree of polymorphism. Still, in theory those loci are molecular markers that can be applied to unravel the forces that maintain genetic diversity and generate population genetic structure in this soil and cave inhabiting species. Furthermore, it seems obvious that the dataset and its accompanying microarray could be helpful in finding out whether transcriptional regulation is an important driver of adaptive evolution in this species.

### Phylogenetics and comparative genomics

Collembola take an exceptional and fascinating position in the tree of life. Together with other basal hexapods (e.g. Protura, Diplura) they are positioned in-between the insects and crustaceans. However, recently some authors suggested that the six-legged body plan found among basal hexapods and insects evolved minimally twice (e.g. [46,47]). The dataset presented here might add the sequence information that is needed to gain a more detailed insight into the evolution of these groups, and the relationship between insects and crustaceans. Using the BLAST tool, Collembase can be queried for genes valuable for phylogenetic inference. Degenerate PCR primers can be developed on the retrieved sequences to obtain information on other basal hexapod groups.

## Conclusion

Collembase provides EST and related data on the springtail *F. candida*. In the near future this database will be supplemented with microarray expression data. We expect that our strategy will impact soil quality testing. In addition, it is clear that Collembase holds information applicable to many fields of ecological sciences (e.g. molecular ecology and ecogenomics, molecular evolution and phylogenetics).

## Availability and requirements

Collembase can be accessed from URL: 

## Competing interests

The author(s) declares that there are no competing interests.

## Authors' contributions

MT participated in the experimental design, prepared the material for the normalized library, performed the bioinformatics analyses and drafted the manuscript. MdB constructed the libraries enriched for stress-responsive genes. TdB, BN and JM assisted in setting up the project, and the laboratory work. RK-L coordinated the sequencing at Greenomics, Wageningen UR. NvS participated in the conception of the study, and helped to draft the manuscript. DR participated in experimental design, supervised the project and shaped the final version of the manuscript. All authors have read and approved the final version of the manuscript.

## Supplementary Material

Additional file 1 The six primer pairs that were used to test the normalization procedure. Click here for file

Additional file 2 Sequence length distribution of different clusters in the assembled dataset. Click here for file

Additional file 3 The clusters that were marked as putative bacterial contamination in Collembase. Click here for file

Additional file 4 Summary of the microsatellite analysis as generated by MISA . Click here for file
