# Supplementary figures and images for: Collembase: a repository for springtail genomics and soil quality assessment
Source: BMC Genomics. 2007 Sep 27;8:341. doi: 10.1186/1471-2164-8-341 (PMC2234260; doi:10.1186/1471-2164-8-341)

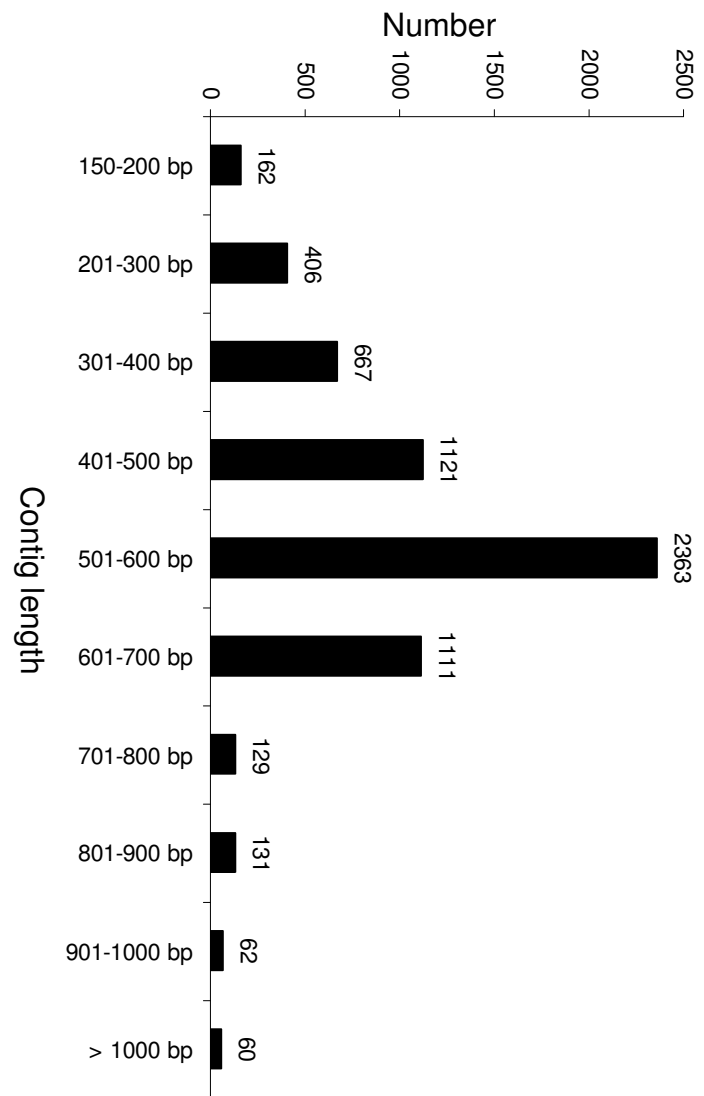

Supplement: Additional file 2 — Sequence length distribution of different clusters in the assembled dataset. [file 1471-2164-8-341-S2.pdf]
