# Supplementary material for: Collembase: a repository for springtail genomics and soil quality assessment
Source: BMC Genomics. 2007 Sep 27;8:341. doi: 10.1186/1471-2164-8-341 (PMC2234260; doi:10.1186/1471-2164-8-341)
Supplement: Additional file 3 — The clusters that were marked as putative bacterial contamination in Collembase. [file 1471-2164-8-341-S3.doc]

Additional file 3: The clusters that were marked as putative bacterial contamination in Collembase.

E. coli:

Fcc00133 Fcc01530 Fcc02390 Fcc03342 Fcc04738 Fcc06119

Fcc00318 Fcc01807 Fcc02567 Fcc03436 Fcc04992 Fcc06126

Fcc00371 Fcc01901 Fcc02674 Fcc03438 Fcc05066 Fcc06161

Fcc00422 Fcc01999 Fcc03004 Fcc03451 Fcc05269 Fcc06244

Fcc00579 Fcc02033 Fcc03039 Fcc03627 Fcc05437 Fcc06254

Fcc00720 Fcc02223 Fcc03216 Fcc03734 Fcc05508 Fcc06273

Fcc00809 Fcc02268 Fcc03217 Fcc03801 Fcc05597

Fcc00824 Fcc02284 Fcc03237 Fcc03916 Fcc05678

Fcc01389 Fcc02329 Fcc03303 Fcc03946 Fcc05794

Fcc01447 Fcc02350 Fcc03330 Fcc04667 Fcc05861

Wolbachia:

Fcc00012 Fcc02170 Fcc04876 Fcc06129

Fcc00133 Fcc02549 Fcc04902 Fcc06244

Fcc00318 Fcc03237 Fcc04992

Fcc00706 Fcc03330 Fcc05066

Fcc00720 Fcc03438 Fcc05111

Fcc00795 Fcc03916 Fcc05437

Fcc01447 Fcc03946 Fcc05508

Fcc01530 Fcc04112 Fcc05638

Fcc01807 Fcc04268 Fcc05794

Fcc02127 Fcc04463 Fcc05861

Overlapping clusters:


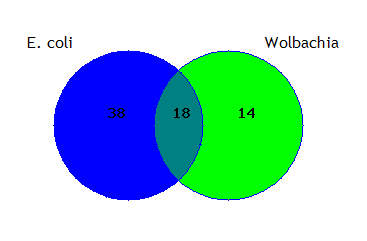


Fcc03237 Fcc00133

Fcc05437 Fcc00720

Fcc05508 Fcc01530

Fcc03916 Fcc01447

Fcc03438 Fcc05861

Fcc03946 Fcc00318

Fcc01807 Fcc04992

Fcc03330 Fcc05066

Fcc05794

Fcc06244
